# Effects of Exercise or Mechanical Stimulation on Bone Development and Bone Repair

**DOI:** 10.1155/2022/5372229

**Published:** 2022-09-28

**Authors:** Lidan Song

**Affiliations:** Institute of Physical Education, Anyang Normal University, Anyang, 455000 Henan, China

## Abstract

The development and regeneration of the bone are tightly regulated by mechanical cues. Multiple cell types, including osteoblasts, osteocytes, osteoclasts, mesenchymal stem cells (MSCs), and recently found skeletal stem cells (SSCs), are responsible for efficient bone development and injury repair. The immune cells in the environment interact with bone cells to maintain homeostasis and facilitate bone regeneration. Investigation of the mechanism by which these cells sense and respond to mechanical signals in bone is fundamental for optimal clinical intervention in bone injury healing. We discuss the effects of exercise programs on fracture healing in animal models and human patients, which encouragingly suggest that carefully designed exercise prescriptions can improve the result of fracture healing during the remodeling phase. However, additional clinical tracing and date accumulation are still required for the pervasive application of exercise prescriptions to improve fracture healing.

## 1. Introduction

The skeleton senses and responds to mechanical signals while maintaining the tissue homeostasis [1]. Mechanical forces take part in regulating the process of bone development, repair, and regeneration by influencing multiple cells in the bone. As it is able to completely recover without the formation of scar, the mechanism of bone regeneration has attracted the attention of scientists and clinicians. The three phases during bone repair, the inflammatory phase, the proliferation phase and remodeling phase, involve various cell types, including neutrophils, macrophages, endothelial cells, osteoblasts, osteoclasts, mesenchymal stem cells, and skeletal stem cells [2]. We review how these components are regulated by mechanical stimulation during the repair processes. In addition to the molecular mechanism of mechanical regulation during bone repair and regeneration, in vivo studies to investigate the effects of exercise on fracture repair are introduced. The results from animal models show that mechanical stimulation during the remodeling phase significantly enhanced the formation of the callus and ultimately promoted fracture repair. We also discuss clinical research that surveyed the effects of exercise on hip fracture recovery. While some of these studies showed no difference between the exercise group and the control group, some found that patients attained better physical performance and quality of life in the exercise group. More clinical data and analysis are needed to increase the prevalence of exercise prescriptions for better recovery of fracture patients. In sum, we describe the mechanical regulation of the bone during bone development, repair, and regeneration, as well as the effects of exercise on fracture repair.

## 2. Homeostasic Maintenance of Bone

The bones in the body can be categorized into four types according to their shapes: long bones, short bones, flat bones, and irregular bones. Bones with specific shapes and anatomical locations function to support the posture and locomotion of the body, protect the viscera and hematopoietic system, and maintain the balance of mineral and secreted cytokines, growth factors, and other factors to execute reciprocal regulation with other parts in the body.

**Figure 1:**
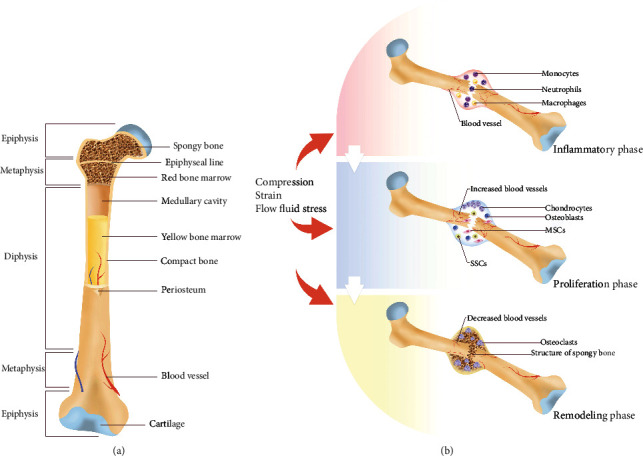
Bone structure and bone repair processes. (a) The basic anatomic structure of long bone. (b) The three phases in bone injury repair.

In long bones, the hollow shaft in the central part of the long bone is the diaphysis, where dense cortical bone dominates with the bone marrow (Figure 1(a)). In the ends of the long bones, the epiphysis above the growth plate is composed mainly of a trabecular meshwork bone lined by a layer of hyaline cartilage. Between the epiphysis and the diaphysis, the region below the growth plate is called the metaphysis [3]. Flat bones, such as skull and rib bones, possess a layer of sponge bone and two layers of compact bone around it.

The cortical bone is covered by periosteum and endosteum outside and inside of the bone cavity [4]. With their specific location and abundant cell types, the connective fibrous tissue, periosteum, and endosteum have been proven to participate in bone regeneration [5] and hematopoietic stem cell population preservation [6].

The maintenance of bone homeostasis is mainly dependent on the equilibrium between bone-forming osteoblasts and bone-resorbing osteoclasts. Other cell types in the skeleton include osteocytes and chondrocytes. For the maturation of osteoblasts, mesenchymal stem cells (MSCs) and skeletal stem cells (SSCs) are needed. First found in the bone marrow, bone marrow mesenchymal stem cells (BM-MSCs) are multipotent stromal cells with the ability to differentiate into osteoblasts, chondrocytes, and adipocytes. MSCs express specific markers, including CD73, CD90, and CD105 [7]. In addition to the bone marrow, MSCs can also be isolated in other sites, including the periosteum and cortical bone [8]. In the recent decade, SSCs have been found in the growth plate and periosteum, whose role in bone regeneration is discussed in the next section. The deficiency of periosteal stem cells leads to impaired postnatal skeletal growth [9]. The relationship between the two stem cell populations in the skeleton has not been clearly expounded beyond the restricted comprehension of the full cell composition. Compared with MSCs, which were found in the bone marrow [10], SSCs sorted through cell surface marker combinations are relatively newly characterized cell populations, the properties and functions of which required further research.

## 3. Bone Development and Tissue Repair Process

### 3.1. The Development of Bone

As the scaffold of the body, the development of the skeleton requires coordinated mobilization of different cells derived from multiple germ layers. Neural crest cells derived from neural ectoderm give rise to part of the craniofacial bones and cartilage in the anterior skull. The formation of the posterior skull is dependent on cells from the prechordal mesoderm. The paraxial mesoderm (somites) is responsible for the formation of the axial skeleton, while cells from the lateral plate mesoderm develop into the appendicular skeleton [11]. Two processes with different cell transitions, intramembranous ossification and endochondral ossification, mediate ultimate bone maturation throughout the body. Intramembranous ossification is found in the development of flat bones, including the skull, mandible, maxilla, and clavicle, during which the mesenchymal cells in the condensation differentiate directly into osteoblasts and osteocytes. On the other hand, endochondral ossification involves the formation of cartilage primordium, where the mesenchymal cells in the center differentiate into chondrocytes first and the perichondrium formed by surrounding chondrocytes compartmentalizes the future bone from other surrounding tissues. The hypertrophy of the chondrocytes in the perichondrium is followed by the invasion of blood vessels, which allows for the recruitment of osteoprogenitors and cartilage-absorptive cells. Then, the bone marrow cavity, which is also called the primary ossification center and the trabecular bone and haematopoietic cells within it arise. Following the continued expansion of the primary ossification center, the secondary ossification centers, which lead to the development of the epiphysial growth plate, form in the ends of the growing bones [12]. Essential for the elongation of long bones, the growth plate is a complex region with chondrocytes at different states [13]. Located close to the epiphysis, chondrocytes in the quiescent zone serve as a pool for proliferating chondrocytes in the proliferative zone. Towards the diaphysis, chondrocytes stop proliferating to become hypertrophic. Some of these cells undergo apoptosis, while the other cells become osteoblasts [14].

### 3.2. Mechanical Stimulation in Bone Development

Normal development of the musculoskeletal system requires precise coordination of bone and skeletal muscle. Except for the cells that differentiate into osteolinage cells, normal development of skeletal muscle is also necessary for the occurrence of functional bones and joints. Muscle force is indispensable for correct musculoskeletal assembly. Aberrant muscle formation in paralyzed mouse embryos hinders the development of the bone by impacting the length of the growth plate and number of proliferating chondrocytes. Joint fusion in mouse embryos was found when muscle contraction was deficient [15]. During adulthood, the regulation of bone homeostasis by mechanical stimulation is more obvious. Mechanical load dynamically affects numerous aspects of bone, including the trabecular bone volume and the thickness of cortical bone [1, 16]. The discovery of the mechanosensitive channel protein Piezo1 partly explained the mechanism by which mechanical stimulation regulates bone formation.

The influence of mechanical loading on bone is not limited to bone cells. Since osteal lineage cells are niche cells of the hematopoietic system, it is rational to hypothesize that the response to mechanical loading of osteal lineage cells affects the hematopoietic cell populations. The effects of acute exercise and long-term exercise training on hematopoietic stem cell (HSC) survival, mobilization, and other characteristics before and after HSC transplantation have been discussed [17, 18]. The rapid development of single-cell RNA sequencing (scRNA-seq) technology and the refinement of cytometry allow for elucidation of the responses of the bone microenvironment, including hematopoietic cells and immune cells, to mechanical stimulation [19].

### 3.3. Bone Fracture Repair and Regeneration

Fracture is a frequent injury occurring in the musculoskeletal system. Some patients undergo delayed repair and nonunion, which severely impact work capability and quality of life. Investigation of the mechanism of bone repair and regeneration may provide more approaches for optimal treatment and more efficient repair. During the occurrence of fracture, the rupture of blood vessels and soft tissues directly leads to the initiation of the first phase of fracture healing, the inflammatory phase [20]. The conversion of fibrinogen into fibrin facilitates the formation of hematoma, where circulating and resident immune cells are recruited by the injury signal. Following the recruitment of neutrophils [21], macrophages invading into the injury site undergo population transition, which changes the state of the healing tissue from proinflammatory to anti-inflammatory by altering the cytokines secreted by the macrophages [22]. Precise temporal and spatial regulation of immune cell behavior, including migration and polarization, is necessary for efficient fracture repair [23]. Then, the presence of lymphocytes in the fracture site activates adaptive immunity for fracture healing [24]. In addition to immune cells, other more environmental cells have been found to regulate the process of bone regeneration. For example, Schwann cells were demonstrated to promote mandibular repair through crosstalk with skeletal stem cells [25].

After the activation and recruitment of MSCs and SSCs and the development of osteogenic progenitor cells, rapid differentiation and proliferation of osteoblasts begin the second phase, the proliferation phase. In this phase, a callus forms to turn the hematoma into a harder scaffold between the broken ends. Through endochondral ossification and intramembranous ossification, the formation of a cartilage callus by newly formed osteons completes the union of the fractured bone (Figure 1(b)).

In addition to immune cells and bone-forming and bone-absorbing cells, endothelial cells that mediate angiogenesis and vasculogenesis are indispensable in bone regeneration. The two processes of new blood formation, angiogenesis and vasculogenesis, involve the development of new blood vessels with or without a preexisting vascular component [26]. Whether both of the processes contribute to fracture healing or whether one of them dominates in the repair is still an open question. However, there is no doubt that active blood vessel formation occurs at the injury site. The blood vessels formed during fracture repair provide the hematoma or callus with oxygen, nutrients, and cells participating in the healing processes, such as MSCs. In addition, the immune cells and MSCs secrete growth factors, including vascular endothelial growth factors (VEGF), to promote the formation of new blood vessels, which also drive repair. Three isoforms of VEGF, A, B, and C, form homo- and heterodimers to regulate the cell behavior of endothelial cells by binding to their receptors, VEGFR1 and VEGFR2 [27]. The differentiation and proliferation of endothelial cells are enhanced by VEGF, which also activates the recruitment and tube formation capacity of endothelial progenitor cells. The collapse of the intact bone and the destruction of the blood supply system result in necrosis of the perifracture tissue and hypoxia in the hematoma and adjacent tissue. By modifying the expression of hypoxia-inducible factor *α* (HIF*α*), it was been discovered that osteoblasts sense the oxygen level and couple osteogenesis and angiogenesis [28]. The expression of VEGF in osteoblasts overexpressing HIF1*α* was upregulated, while the long bones were dense and highly vascularized. When HIF1*α* was deficient in osteoblasts, a reverse phenotype of thinner and less vascularized long bones were observed. This research revealed that correctly regulated angiogenesis is crucial for bone formation and homeostasis maintenance. The fact that growth factors from osteoblasts influence the behavior of blood vessel-forming endothelial cells emphasizes the importance of cell interactions in tissue repair. When normal blood supply cannot be met, pathological cases are present [29]. Patients with abnormal distal arteriograms face a higher risk of nonunion. Impaired vascular in-growth to the callus in open fracture also increased the risk of nonunion, more tissue necrosis and reduced resistance to infection [30]. Interestingly, in the observed correlation between smoking and increased risk of fracture, it was hypothesized that smoking impedes vascularization at the fracture healing site by the action of nicotine, thus leading to delayed mineralization and unrepaired bone fracture [31]. Although public health data show that the rate of smoking in patients with tibial nonunions is higher than that in the general public [32], more evidence, including mechanistic research, is needed.

As one of the determinants of successful bone regeneration, vascularization is a target for the application of tissue engineering in bone repair improvement. The combination of VEGF and materials for bone regeneration enhancement increased blood infiltration and bone mineral density in in vivo bone defect models [33, 34].

After robust osteogenesis in the proliferation phase, the shape of the bone differs from that prior to injury, which is why osteoclasts are needed to start the remodeling phase. Recruited by receptor activator of nuclear factor­*κ*B ligand- (RANKL-) expressing osteocytes, osteoclast precursors give rise to mature osteoclasts, which function in the resorption of the redundant bone [35]. Correct progress of these healing phases guarantees that the bone can be repaired to its uninjured form without the formation of a scar.

### 3.4. Stem Cells in Bone Regeneration

Given the potent multidirectional differentiation capacity of stem cells and the attractive prospects of their clinical application, the exploration of stem cells of specific tissues has not stopped since the first discovery of hematopoietic stem cells [36]. During the past decade, human and mouse skeletal stem and progenitor cell populations and their hierarchy have been identified by a combination of specific cell surface markers [37, 38]. The cell surface marker combination was determined according to the information from single-cell RNA-seq, which examined skeletal tissue cells. The differentiation potential of the populations was verified through in vitro differentiation and kidney capsule injection experiments. The definition of skeletal stem cells is not restricted to a single surface marker combination; other common molecular markers such as Ctsk [39] and Gremlin1 [40] have been found to mark a specific stem cell population. Given the diversity of the cells in bone tissue, the development of different regions is thought to be dependent on the skeletal stem/progenitor cells from corresponding locations, which has been demonstrated by evidence from lineage tracing experiments. Among the various parts, the growth plate [41] and periosteum [42] have received particular attention due to their importance for bone growth and regeneration.

Since the identification of skeletal stem cells, their participation and function in bone development, regeneration, aging, and bone-related diseases have been gradually unveiled. Gli1 was found to identify a cell population residing beneath the growth plate that produces osteoblasts during bone development and fracture repair [43]. Through the utilization of lineage tracing and cell lineage analysis, parathyroid hormone-related protein- (PTHrP-) expressing chondrocytes in the rest zone of the growth plate were identified as a population of skeletal stem cells that express skeletal stem cell surface markers and give rise to the hypertrophic chondrocytes of the growth plate [44]. The same research noted that Indian hedgehog (Ihh) signaling is involved in the preservation of this growth plate skeletal stem cell population. The SSCs identified in mice through the immunophenotype (CD45−TER119−Tie2−AlphaV+Thy−6C3−CD105+) were found to expand because of the initiation of the fracture repair process and mediate bone formation during healing [45]. The SSC population marked by the cell marker Ctsk was demonstrated to take part in fracture bone formation via intramembranous ossification [39]. Transcriptome analysis of this periosteal SSC distinguished it from other skeletal stem cell populations that mediate bone formation through endochondral ossification, which indicates the complexity and diversity of the SSC populations and their functional pathways in bones. In another study, the SSC population found in the periosteum, labeled by Mx1 and *α*SMA, was proven to be responsible for the generation of periosteal osteoblasts. Rapid migration of these cells to bone injury site was observed, mediated by CCL5 and its receptors CCR3 and CCR5 [46]. The discovery of the mechanism regulating the migration behavior of SSCs sheds new light on the mobilization of SSCs in bone regeneration.

Additionally, the bone marrow is a complicated environment where elaborately regulated bone cell and hematopoietic cell interactions occur. With the continuous innovation of the methods used to portray the cell populations in tissues, it is not hard to imagine that more markers will be proposed in future investigations. For instance, the invention of spatial single-cell transcriptomics, which adds spatial information to single-cell transcriptomics, significantly deepened the comprehension of tissue development and regeneration [47]. The application of this cutting-edge technology may provide new information and concepts about skeletal stem cells.

## 4. The Role of Mechanical Stimulation in Bone Repair and Regeneration

### 4.1. Mechanical Stimulation in Bone Repair

**Figure 2:**
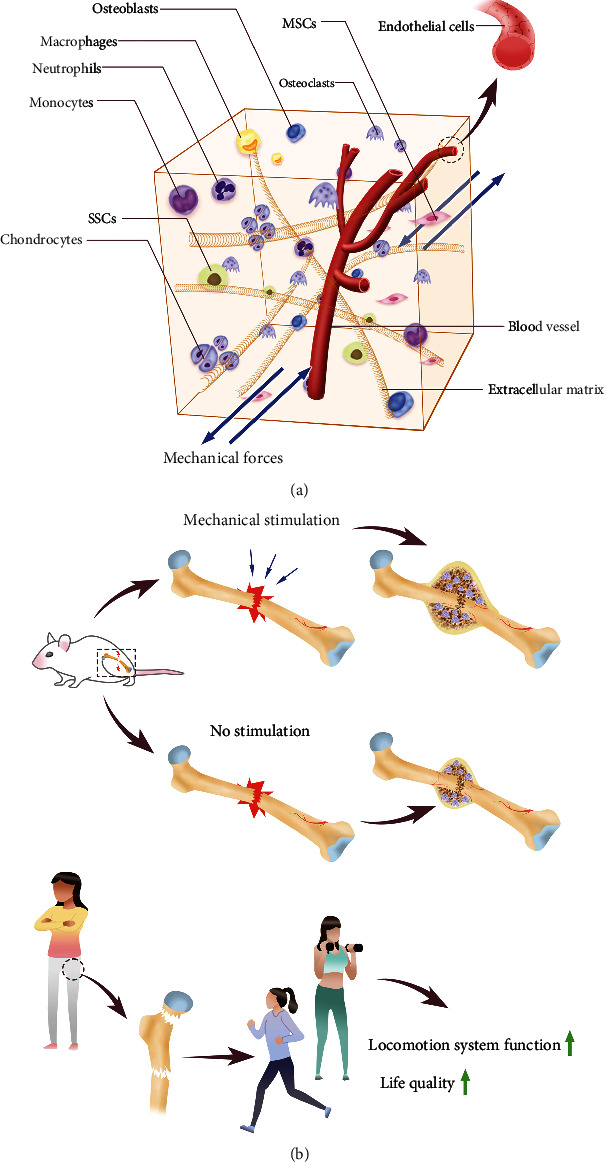
Mechanical stimulation and bone repair. (a) Cells and ECM in the bone tissue receive mechanical signals. (b) The effects of mechanical stimulation and exercise on bone injury repair.

In addition to the resolution of inflammation, a stable supply of the required growth factors and appropriate mechanical stimulation are necessary for bone fracture healing from the very beginning of the repair process (Figure 2(a)).

Immediately after the fracture, the fixation of the broken bone will assure normal callus formation and eventual ossification. It has been proposed that rigid fixation mainly results in intramembranous ossification, while flexible fixation induces the process of endochondral ossification. Apparently, the mechanical strain in the fixed space affects the bone formation fashion and velocity to a large extent. During the proliferation and long-lasting remodeling phase, mechanical stimulation with proper intensity and frequency is beneficial to increase bone formation at the fracture site. Fundamentally, the influence of mechanical fixation and loading on bone regeneration is attributed to the response of the cells that function during the process and the extracellular environment and mechanotransduction between them. Through the three stages of sensation of the mechanical signal, signal transduction, and the response stage, mechanical stimulation affects the shape of the normal and injured bone. To investigate the mechanism by which the different types of cells respond to mechanical signals, various in vitro systems were used to simulate mechanical stimulation. Oscillatory fluid flow, shear stress, fluid pulse, compression, and stretch with different intensities and frequencies have been utilized to determine the response of cells and explore relevant molecules and signaling pathways.

### 4.2. Mechanical Regulation of the Cells in Bone

Dwelling in the lacunae, the osteocytes construct a subtle network to communicate with each other and function as mechanosensors [48]. The stress, strain, and shear fluid stress transmit mechanical signals to the osteocytes. Among the mechanical sensing proteins, Connexin43 (Cx43) has attracted significant attention as it allows for the formation of gap junctions between osteocytes and the transmission of signals, as a hemichannel protein [49]. Increased expression of Cx43 and material exchange were found in osteocytes after shear fluid stress stimulation. Structurally, it has been reported that the application of shear stress on the dendritic side of osteocytes results in the opening of hemichannels in the cells. Moreover, other mechanosensitive proteins, including transient receptor potential vanilloid 4 (TRPV4) [50] and Piezo1, have been found to mediate mechanical stimulation sensation in osteoblasts, osteocytes, and many other cells. Both TRPV4 and Piezo1 are calcium ion channels that mediate the extracellular-intracellular signal transduction through the influx of calcium ions. The Wnt/*β* catenin pathway and extracellular signal regulated kinase (ERK) pathway have been demonstrated to mediate the mechanical signal transduction in osteocytes [51]. In the response stage, multiple factors are produced by the stimulated osteocytes to contribute to the mechanical environment adaptation of the bone. The production of PGE2 in osteocytes, which is mediated by Cox2, accelerated bone formation [52]. However, the expression of sclerostin, an inhibitor of bone formation that antagonizes Wnt signaling, is reduced by oscillatory fluid flow stress.

Of the diverse biophysical cues that regulate the lineage commitment of mesenchymal stem cells, mechanical force is indispensable for the maintenance of bone homeostasis [53]. The conclusion that the stiffness of the extracellular matrix substrate directs mesenchymal stem cell lineage specification in cell culture provides the theoretical basis for the application of material bioengineering in tissue repair [54]. A stiffer extracellular environment induces the differentiation of osteoblasts, while a softer substrate leads to the development of adipocytes and neural cells. RhoA/ROCK signaling [55] and YAP/TAZ signaling [56] have been investigated in the mechanical regulation of mesenchymal stem cells. Although the cell population identification and regeneration participation of skeletal stem cells have been investigated during the past decade, how the mechanical stimulation regulates the cell behavior of SSCs is an important question that still requires to further investigation.

### 4.3. Mechanical Stimulation and Vascularization in Bone Repair

The mechanical regulation of vascularization also suggests the importance of mechanical stimulation in bone regeneration. During bone growth, active vascularization is needed. Mechanical loading of the anterior limbs of rats increased the vascularization in the periosteum [57]. A recent study reported that mechanical forces, which are associated with increased body weight at the end of adolescence, drove the differentiation of the highly angiogenic blood vessel subtype, type H vessels, into quiescent type L endothelium. The transformation of blood vessels hinders the growth of bones [58]. In a rat large bone defect model using compliant fixation plates that allow for transfer of mechanical loads or stiff fixation, early mechanical loading inhibited vascular invasion and bone formation, whereas late (after stiff fixation for 4 weeks) mechanical loading significantly stimulated vascular remodeling and bone regeneration [59]. This study highlights the mechanosensitivity of the vascular network; the evidence showed that the response of the blood vessel network to mechanical forces significantly influences bone growth and regeneration. Given the importance of vascularization in osteogenesis, researchers have tried to address the relationship between physical exercise and angiogenesis during osteogenesis. Mice and rats that underwent treadmill training had significantly larger circulating blood volumes than the sedentary control group [60]. Another study also demonstrated the adaptation of vascularization to mechanical stimulation. Rats that performed running exercise for 2 weeks had a larger number of blood vessels in the tibial proximal metaphysis and higher expression of VEGF receptor mRNA [61].

Although works investigating the effects of exercise on global vascularization have shown that exercise increases the circulating endothelial progenitor cells and angiogenic factors, specific studies on the response of angiogenesis to physical exercise during osteogenesis in humans are still lacking. New technology that allows for noninvasion monitoring of angiogenesis in patients may provide the necessary clinical data to understand how mechanical loading regulates angiogenesis and osteogenesis [62].

## 5. Effects of Physical Exercise on Bone Repair

Although cellular-level research is important to elucidate the molecular mechanism of mechanical regulation during bone regeneration, in vivo studies utilizing various bone injury and regeneration models are necessary to verify the proposed mechanism and assess the clinical implications of the interferences derived from mechanistic research. Additionally, since it is not just about the individual cell behavior and cell crosstalk and interaction are involved, an in vivo study would expand our horizon for an integrative understanding of view of the bone repair and regeneration process. Recently, environmental cells, such as immune cells, endothelial cells, and pericytes, have been recognized to cooperate with tissue cells to facilitate tissue repair [63–66].

In a study using a rat fracture model, the fractured femora were mechanically stimulated 3 times a week between Day 7 and Day 18 postinjury. They found that intermittent tensile strain stimulation during fracture healing promoted chondrogenesis and had better effects on fracture repair than compressive strain or lack of stimulation, which was the control [67]. In another study using rats, approximately 6 mm defects were created in the femora. After the injury, the bones were rigidly fixed by stiff plates or compliant plates that allowed for compressive loading. Examination of the repair by microcomputed tomography, mechanical testing, and histology showed that loading significantly increased the human bone morphogenetic protein-2- (rhBMP-2-) induced regenerated bone volume [68]. Similarly, the femur defect in rats was found to be nonunion without BMP2 completed the repair efficiently. Mechanical loading enhanced the effectiveness of BMP2 in promoting bone regeneration [69]. Using time lapse in vivo imaging, this research indicated that cyclic mechanical loading significantly increased the volume of the mineralized callus of the defective bone during the remodeling phase, which is associated with the regulation of Wnt signaling [70].

The above studies assessing the effects of mechanical stimulation on fracture healing used specific animal models and surgery methods and, more importantly, customized designs for the mechanical stimulation. Different stimulation methods may lead to varying or even opposite conclusions [71]. Although mechanical stimulation realized by machine resulted in improved callus properties and healing efficiency, a study testing the effects of exercise on fracture healing in a mouse model failed to detect any significant difference between the exercise group and the control group in bone fracture with stable fixation [72]. It is possible that an adjusted exercise program or injury method would lead to different results. Therefore, it is prudent to learn the specific experimental parameters when evaluating the clinical implications of basic research. Furthermore, the surgery causing bone injury and the mechanical stimulation method require unification for more efficient and reliable communication of research achievements.

According to the encouraging results from the basic research based on animal models described above, it seems that mechanical stimulation during remodeling can be beneficial for human fracture healing (Figure 2(b)). An elaborately designed and adjusted exercise prescription can benefit patients with musculoskeletal problems. In regard to the clinical effects of exercise on bone injury repair and regeneration, the research results we can review at present are mainly concerned of the application of exercise in fracture recovery, especially for populations with impaired bone formation capacity, such as older and menopausal women. Fracture healing for most young patients is easier than for older patients because of the more exuberant bone formation capacity. Physical activity usually returns to the normal level prior to injury [73].

In the Baltimore hip study experience, women 65 years of age and older who underwent hip fracture were recruited to participate in a home-based postfracture exercise program, which included strength and aerobic components and expected the patients to exercise for 5 days per week. Although this study did not determine whether exercise improved the hip fracture healing of these frail older women, the survey showed that a home-based exercise program of strength and aerobic training after hip fracture is feasible for older patients [74]. In a randomized controlled study involving 26 older adults who experienced hip fracture, patients in the exercise group received short-term leg-strengthening exercise arranged by physical therapists, while the control group received subcutaneous electrical nerve stimulation and mental imagery. The exercise intervention was exerted twice a week for 10 weeks. Through measurements including isometric force production of lower extremity muscles, usual and fast gait speed, and a modified physical performance test etc., the study concluded that the short-term, high-intensity exercise improved the strength, walking ability, and locomotion system function of the patients compared to the control group 1 year after hip fracture [75]. A study with 33 postmenopausal women engaged in 3 months of weight-bearing and resistance training showed that exercise significantly increased the amount of osteogenic marker pro-collagen type 1 N-terminal peptide (P1NP) and circulating osteogenic cells and improved the quality of life [76]. For older hip fracture patients, a 12-month home-based exercise program intervention was also shown to improve the functioning and physical performance of the subjects compared to the patients in the control group who received the usual care only.

However, the actual situation can be more complicated than the causal relationship that physical exercise therapy improves the performance of fracture patients. There are also examinations reporting no obvious effects of physical exercise training on fracture rehabilitation. In a study that recruited 32 control and 38 intervention volunteers aged 65 years or older and had just undergone hip fracture, the intervention group received supervised high-intensity exercise training twice a week for 8 weeks. Through assessments including a one repetition maximum (1RM) test for muscle strength evaluation, a 6-minute walk test, timed up and go test, functional reach test, and observational gait analysis, they did not find significant differences between the control and intervention groups. Another randomized controlled trial recruited 124 patients who had received surgery repair of a hip fracture and gave the intervention group a twelve-month, high-intensity progressive resistance training [77]. Through the evaluation of mortality, nursing home admissions, basic and instrumental activities of daily living (ADLs), and assistive device utilization, they concluded that high-intensity weight-lifting exercise training reduced the risk of death and nursing home admissions of hip fracture patients in the intervention group. Moreover, the basic ADLs declined less and assistive device use was reduced in the intervention group compared with the controls. In this research, exercise significantly exerted positive effects on the subjects' recovery from hip fracture. From the assessment results provided, it seems that exercise with a specific intensity that lasts for a long time can improve the quality of life of fracture patients. It is suggested that even for elderly individuals, receiving treatment for fracture, appropriate exercise training after fracture can be recommended instead of long-time inactivity. The two cases above show that it is still not feasible to directly compare the results from different clinical trials, since the exercise protocols and evaluation methods utilized can be fairly different. A study to assess the effects of weight-bearing and nonweight-bearing exercise on hip fracture rehabilitation recruited 80 inpatients who had suffered from fall-related hip fracture. The subjects were divided into two groups that received weight-bearing or nonweight-bearing exercise prescribed by a physiotherapist for 2 weeks. Strength, balance, gait, and functional performance were evaluated in the two groups. There was little difference in the improvements after receiving the two forms of exercise therapy. In this specific trial, it seems that weight bearing is not a key factor that influences the effectiveness of exercise. However, the exercise time in this case was relatively short compared with other trials that lasted for 1 year or longer. Thus, it is difficult to conclude if weight-bearing exercise lasting for a longer time would result in different outcomes.

Notwithstanding the limitations in these clinical studies in determining the effects of exercise on fracture healing, the results suggest that appropriate exercise prescriptions made by professional physical therapists can effectively improve the locomotion capability and quality of life of patients. Supervision of the exercise exertion and the tracing of the postexercise data are important to help clinicians to optimize exercise programs for fracture patients [78].

## 6. Conclusion

In this review, we discussed the regulation of bone development and regeneration by mechanical signals and the mechanotransduction of bone cells. As the most researched cell types, osteocytes and mesenchymal stem cells sense mechanical signals and responses and influence the balance of bone formation in healthy and pathological situations. How the mechanical response of the newly discovered skeletal stem cells influences bone regeneration is an intriguing question to explore. The molecular and cellular investigations depict the fundamental signaling pathways involved in the mechanical regulation of the bone, while the studies using animal models directly examined the effects of mechanical loading on fracture healing. The current evidence indicates that mechanical loading is positive for better callus properties and faster bone regeneration. Clinical trials involving older fracture patients showed improved healing and locomotion system function. Improved comprehension of the mechanical regulation of bone tissue and clinical data about exercise intervention influencing fracture healing are required to develop effective fracture treatment.
